# “Close to the teacher, believe in the teaching”: the advantageous effect of interpersonal emotion regulation and the role of teacher-student closeness

**DOI:** 10.3389/fpsyg.2026.1745339

**Published:** 2026-02-04

**Authors:** Wenmei Sun, Xubo Liu, Sasa Ding, Daixin He, Qiaoyu Wu, Shang Li

**Affiliations:** Faculty of Education, Henan Normal University, Xinxiang City, Henan Province, China

**Keywords:** cognitive reappraisal, expressive suppression, interaction, interpersonal emotion regulation, teacher-student closeness

## Abstract

**Background:**

Interaction is a key element in sustaining the development of human society. The Social Baseline Theory suggests that individuals can regulate emotions through social interactions, thereby promoting physical and mental wellbeing.

**Aim:**

This study focuses on the interaction between graduate students and their supervisors within the educational ecosystem, exploring the advantages of interpersonal emotion regulation and the role of teacher-student closeness.

**Methods:**

Study 1 recruited 9 university teachers and 58 graduate students, and Study 2 62 of each, with behavioral experiments implemented in both.

**Results:**

The results indicate that, whether employing cognitive reappraisal or expressive suppression strategies, the effectiveness of interpersonal emotion regulation is significantly superior to that of self-emotion regulation. Moreover, higher levels of teacher-student closeness enhance the positive effects of interpersonal emotion regulation on graduate students.

**Conclusion:**

These findings highlight the advantage of interpersonal emotion regulation in teacher-student interactions and emphasize the critical role of high closeness. This study not only provides empirical support for understanding the effectiveness of interpersonal emotion regulation in teacher-student interactions but also offers important theoretical foundations and practical implications for constructing a mental health support system for graduate students.

## 1 Introduction

Joy, anger, sorrow, and happiness are common human experiences, with diverse emotional states bringing varied feelings to individuals‘ lives (Xiong and Li, 2024). In real life, individuals not only experience emotions alone, but also frequently achieve socially regulated emotions by sharing and confiding in others. Correspondingly, individuals may also become regulators of others' emotions (Xiong and Li, 2024). This bidirectional interaction and mutual influence process reflects the core mechanism of interpersonal emotion regulation.

In recent years, significant progress has been made in emotion regulation research, especially producing numerous discoveries about how people independently regulate their emotional states (i.e., self-emotion regulation). However, scholars have increasingly recognized that understanding how individuals mutually regulate emotions is equally important (Reeck et al., 2016). Studies have shown that a higher tendency and efficacy in interpersonal emotion regulation help individuals establish broader social support relationships (Williams et al., 2018). Additionally, interpersonal emotion regulation can positively predict students' positive academic emotions and enhance their well-being (Wang et al., 2022; Niven et al., 2012; Hu et al., 2020).

As affective social beings, humans rely on interpersonal bonds for psychological adaptation and development (Luo, 2021; Zhu et al., 2025). The graduate stage represents a critical transitional period for individuals' academic and psychological development. Confronted with multiple stressors, graduate students' psychological adaptation is highly dependent on core interpersonal support. This issue is a global concern. International studies have shown that the proportion of graduate students experiencing psychological distress is significantly higher than that of the general population (Allen et al., 2022; Casey et al., 2022), and they also face an elevated risk of developing common mental disorders (Moss et al., 2022). Cross-cultural evidence further confirms its pervasiveness and identifies key influencing factors: A study based on global survey data from Nature revealed that graduate students in multiple countries—including the United States, Australia, China, and Japan—face substantial mental health risks, with social factors (e.g., external relationships, teacher-student interactions) acting as critical variables (Tao and Qie, 2025). Specifically, the role of supervisors is particularly prominent: Research has found that supervisors' negative feedback (e.g., harsh criticism) and unreasonable expectations are important triggers for students' anxiety and depression (Busch et al., 2024), while dysfunctional teacher-student relationships themselves directly impair mental health (SenthilKumar et al., 2023). Collectively, these findings demonstrate that supervisors and the teacher-student relationship are globally critical variables influencing graduate students' mental health.

With the intensification of social competition, the mental health issues of Chinese graduate students have become increasingly severe (Yu and Wang, 2024). Among various influencing factors, the role of supervisors is particularly critical (Gao et al., 2024), which is deeply rooted in the “tutor responsibility system” of graduate education in China. This system establishes supervisors as the core “primary persons responsible” for training, rendering the teacher-student relationship the most fundamental and intimate interpersonal bond during the graduate stage (Liu and Lu, 2025; Chu et al., 2021; Xin et al., 2023). In practice, the “tutor responsibility system” means that supervisors participate in-depth in all aspects of training throughout the entire process and hold critical influence over academic evaluation, research recognition, and even graduation progress (Jiang et al., 2022). This model, combining “full-process participation” and “key evaluation,” leads to graduate students' high level of dependence on their supervisors for academic and personal development. Consequently, the supervisor's role transcends mere academic guidance, extending to multiple dimensions of students' growth and exerting a profound impact on their mental health.

The teacher-student relationship is inherently a continuous and dynamic interactive process. Within this relationship, as the dominant party, supervisors' interpersonal emotion regulation behaviors (i.e., the process of proactively regulating students' emotions) are directly associated with the quality of graduate students' emotional adaptation and serve as a key entry point for mental health interventions (Jin et al., 2025). However, in this specific context, the efficacy advantage of interpersonal emotion regulation over intrapersonal emotion regulation remains unclear, and the pathways for its effective improvement await further exploration (Wang J. et al., 2024). Therefore, integrating the Chinese cultural context and teacher-student relationship characteristics, a systematic exploration of supervisors' interpersonal emotion regulation (its effects and mechanisms) holds significant theoretical and practical value for easing Chinese graduate students' psychological distress and promoting their positive development.

Interpersonal Emotion Regulation (IER), refers to the process by which an individual (the regulator) consciously attempts to alter another person's (the target's) emotional responses (Reeck et al., 2016). Its essential characteristic lies in its social interactivity (Hu et al., 2020). Interaction is one of the core elements of human social functioning (Redcay and Schilbach, 2019) and is particularly important when individuals cope with negative emotions. Appraisal Theories of Emotion posit that emotions arise from an individual's cognitive evaluation of a situation or event, and others can play a role in this process (Fischer et al., 2016; Zhao and Bai, 2025). The Social Baseline Theory (SBT) further proposes that the presence of others offers individuals the possibility to regulate emotions through social interactions, thereby effectively conserving mental and physical resources (Beckes and Coan, 2011).

Existing studies have shown that, compared to facing emotional situations alone, the presence of others can significantly reduce individuals' negative emotional experiences (Xie et al., 2023; Yao et al., 2025). A study involving romantic couples found that IER was more efficient in comparison to self-emotion regulation in alleviating individuals' distress (Levy-Gigi and Shamay-Tsoory, 2017). In family settings, parents' emotion regulation toward their children facilitates the growth of the children's self-regulation abilities (Reeck et al., 2016; Franklin-Gillette and Shamay-Tsoory, 2021). The aforementioned research provides empirical support for the advantageous effects of IER across different contexts. The interaction between graduate students and supervisors, characterized by high frequency and depth, lays the foundation for interpersonal emotion regulation in educational settings. Therefore, we hypothesize that a similar regulatory advantage may exist in the educational domain.

Emotion regulation strategies are the core mechanism for achieving emotion regulation effects (Huang et al., 2024). Of these, cognitive reappraisal (CR) and expressive suppression (ES)—the two most representative types—have been widely studied. CR modifies emotional experience by changing cognitive interpretations of emotional events, while ES involves inhibiting ongoing emotional expressive behaviors (Zhang et al., 2022).

In research on self-emotion regulation, multiple pieces of evidence indicate that CR is more effective than ES (Gross, 2002). Specifically, CR is an antecedent-focused strategy that involves proactively reconstructing events positively or rationally before emotional responses occur, thereby reducing adverse emotional experiences and expressions. In contrast, ES is a response-focused strategy that, while capable of inhibiting or suppressing outward emotional behaviors, struggles to effectively alleviate internal emotional experiences and physiological arousal (Zhang S. et al., 2024). Compared to expressive suppression, cognitive reappraisal significantly reduces negative emotional experiences and behavioral expressions (Ma et al., 2010), with particularly notable advantages in diminishing negative emotional experiences (Yan et al., 2022).

Compared to the relatively systematic research on CR and ES in the field of self-emotion regulation, empirical studies in the domain of interpersonal emotion regulation remain limited. Existing research has found that although both strategies can, to some extent, reduce the negative emotional experiences of the regulated individual, the regulated individuals rate the success of CR strategies markedly higher in comparison to that of ES (Liu et al., 2023). Furthermore, CR outperforms ES in enhancing the pleasantness of the regulated individual (Zhang, 2023; Zhu et al., 2025). More importantly, studies on interpersonal communication indicate that expressive suppression can disrupt the communication process and potentially harm interpersonal rapport (Butler et al., 2003). In summary, whether in the context of self-emotion regulation or interpersonal emotion regulation, cognitive reappraisal demonstrates superior efficacy compared to expressive suppression.

Although humans have developed diverse strategies to cope with negative emotions, individuals still exhibit a significant tendency to seek comfort from significant others when facing distressing situations (Levy-Gigi and Shamay-Tsoory, 2017). Social relationships are closely linked to individuals' physical and mental health (Marroquín et al., 2017). Similarly, emotional wellbeing also largely depends on the intimate relationships one possesses (Zaki, 2020). The Interpersonal Needs Theory provides a theoretical basis for this phenomenon, indicating that the need for relationships is one of the core psychological demands of humans, and closeness is a key element in fulfilling this need (Wang S. et al., 2024).

Empirical research further supports the critical role of closeness in interpersonal emotion regulation. Studies indicate that merely the presence of close others can effectively reduce individuals‘ emotional responses in stressful and distressing situations (Beckes and Coan, 2011; Zaki, 2020). Because close others possess an in-depth understanding of an individual's life, personality, and behavioral patterns, they are often more effective in improving the individual's perception of emotional issues (Thompson et al., 2024). For example, in a study where negative emotional images were used to induce negative emotions, participants achieved significantly better emotion regulation outcomes with the assistance of friends compared to regulating alone or with the help of strangers (Morawetz et al., 2021). Similarly, research focusing on adolescent populations provides strong evidence that only under conditions of closer interpersonal distance can regulators' cognitive empathy enhance emotion regulation effectiveness by improving the accuracy of their judgments regarding the target's emotional state (Wang and Su, 2023). This phenomenon has also been validated in marital and close friendship contexts, demonstrating that higher interpersonal closeness leads to more significant interpersonal emotion regulation outcomes (Zhang et al., 2023; Yao et al., 2025).

For graduate students, the teacher-student relationship constitutes an important external environmental resource during their academic tenure, which can serve a buffering and protective role when confronting negative academic and life events (Xin et al., 2023). A longitudinal study revealed that a positive supervisor-student relationship contributes to enhancing graduate students' subjective wellbeing and positive psychological welfare (Liang et al., 2021). Moreover, the higher the perceived closeness between students and teachers, the stronger the positive emotions experienced by students (Goetz et al., 2021).

Drawing on the above conceptual and evidence-based findings, this study hypothesizes that in the process of interpersonal emotion regulation, conditions of high teacher-student closeness are more conducive to alleviating negative emotions among graduate students. This is compared to conditions of low teacher-student closeness.

In summary, this study primarily addresses two questions: (1) Does interpersonal emotion regulation demonstrate an advantageous effect? (2) Does teacher-student closeness influence the effectiveness of interpersonal emotion regulation among graduate students? Based on these questions, this paper will employ two behavioral experiments to systematically investigate these issues.

## 2 Study 1: the impact of emotion regulation types and strategies on emotion regulation effectiveness among graduate students

### 2.1 Participants

Sample size calculation was performed via G^*^Power 3.1 (Faul et al., 2009). With an *effect size* of *0.25*, α set to *0.05*, and *1-*β at *0.95*, the analysis showed 54 participants were needed.

Based on this estimation, this study randomly recruited participants from a university, specifically including both graduate students and university teachers. A total of 58 graduate students were recruited (including 18 males and 40 females, *M*_*age*_ ± *SD* =25 ± 1.99). Following the approach of Zheng et al. (2018), we also recruited 9 university teachers (including 2 males and 7 females, *M*_*age*_ ± *SD*= 37.44 ± 4.86). All involved individuals possessed normal vision or vision corrected to normal, with no neurological conditions present.

Before the trial began, the investigator fully outlined the study's goals, steps, possible hazards, and participant entitlements—including the freedom to withdraw at any time and data privacy protections—to each participant. After ensuring full comprehension, participants voluntarily signed a paper-based informed consent form. The present study has been sanctioned by the institutional Academic Committee.

### 2.2 Measures

#### 2.2.1 Emotional picture stimuli

The images were sourced from the GAPED (www.affective-sciences.org/researchmaterial) (Dan-Glauser and Scherer, 2011), a standardized database widely used in international emotion psychology research, providing multidimensional ratings of emotional valence and arousal. Initially, 80 negative images were randomly selected from the database. Given that the original ratings in GAPED were based on Western participants, cultural differences might lead to deviations in emotional perception among Chinese participants. Therefore, a localized emotional valence assessment was conducted prior to the formal experiment. The specific procedure was as follows: An additional 45 students who were not involved in the core experiment were asked to assess the images' valence and arousal using a 9-point rating scale (Xu et al., 2021). After excluding images with higher valence ratings, 74 images were retained for statistical analysis. The results showed: *M*_*Valence*_ ± *SD* = 2.64 ± 0.25; *M*_*Arousal*_ ± *SD* = 6.31 ± 0.34. Based on the scale range, these images effectively induced negative emotional experiences, meeting the experimental requirements for emotional stimuli. The 74 images were then randomly divided into two groups (cognitive reappraisal group and expressive suppression group), *t-tests* confirmed no statistically significant differences (*p*_*valence*_ = *0.67;p*_*arousal*_ = *0.061*) between the two groups.

#### 2.2.2 Emotion regulation strategy sentence materials

Referring to the sentence framework used in the experimental procedure of Liu et al. (2023) and incorporating the core characteristics of the negative emotional stimuli in this study, 8 CR strategy sentences and 8 ES strategy sentences were collaboratively developed with two psychology experts (e.g., “This is just a picture, not real,” “Please smile and avoid dwelling on your negative emotions…”). The sentences were designed to be concise, clearly directive, and aligned with the linguistic habits of Chinese participants. Subsequently, 30 students who were not involved in the core experiment were asked to assess the effectiveness of the two sets of sentences (CR and ES) on a 7-point scale (1 = completely ineffective, 7 = highly effective). The outcomes indicated that the mean scores for individual expressive suppression sentences ranged from 4.53 to 5.13, with an overall mean score of 4.84 ± 0.23 (*M* ± *SD*). The average scores for individual cognitive reappraisal sentences ranged from 4.27 to 4.87, with an overall mean score of 4.53 ± 0.23 (*M* ± *SD*). The mean scores for both sets of sentences were above the median value of 4, indicating that both cognitive reappraisal and expressive suppression sentences effectively guided participants to execute the corresponding emotion regulation behaviors (Xu et al., 2021).

#### 2.2.3 Positive and Negative Affect Schedule (PANAS)

To control for the potential influence of participants‘ baseline emotional states on the experimental results, the PANAS (Watson et al., 1988) was administered before the experiment to assess participants' pre-experiment emotional states. The scale comprises two dimensions: Positive Affect (PA) and Negative Affect (NA), comprising 20 items in total. Participants rated each item on a 1-5 scale (1 = very slightly or not at all, 5 = extremely). The PANAS has shown strong reliability and validity in Chinese populations (Huang et al., 2003). In this study, the Cronbach's α for the Positive Affect subscale was 0.81, and for the Negative Affect subscale, it was 0.78.

#### 2.2.4 Negative emotion rating scale

The Negative Emotion Rating Scale, adapted from the scale used by Yao et al. (2025), was employed to subjectively assess participants‘ emotional valence. This scale utilizes a 1-9 point rating system (1 = extremely unpleasant, 9=extremely pleasant). Focused on the core valence dimension of negative emotional experiences, the scale features concise items and intuitive scoring criteria. It has been applied in multiple domestic studies on negative emotion induction (Yao et al., 2025; Yao and Wang, 2023) and effectively captures subtle changes in participants' emotional valence, making it suitable for the immediate assessment of emotion regulation effects in this study.

#### 2.2.5 Emotion Regulation Questionnaire (ERQ)

The ERQ, developed by Gross and John (2003), was used to assess individual differences in participants' habitual use of CR and ES strategies. The questionnaire consists of 10 items: Items 1, 3, 5, 7, 8, and 10 measure CR, while items 2, 4, 6, and 9 measure ES. Each item was evaluated by participants using a 7-point Likert scale, where 1 stood for “strongly disagree” and 7 for “strongly agree.” The Chinese adaptation of this questionnaire has been proven to have good reliability and validity (Sun et al., 2020). In the current research, the Cronbach's α for the CR subscale was 0.80, while that for the ES subscale reached 0.79.

### 2.3 Experimental design

2 × 2 mixed experimental design was adopted, with emotion regulation strategy (CR vs. ES) as the within-subjects variable and emotion regulation type (self vs. interpersonal) as the between-subjects variable. The dependent variable was the self-reported negative emotion ratings of the students.

### 2.4 Experimental procedure

Ahead of the formal trial, participants were assigned to groups. Twenty-nine graduate students were allocated to the self-emotion regulation group, while another 29 graduate students and 9 university teachers were paired to form “teacher-student dyads” and assigned to the interpersonal emotion regulation group. Upon arriving at the laboratory, participants were guided by the experimenter to complete questionnaires (all participants completed them separately in individual rooms). They were informed of the confidentiality principle, ensuring that personal information and experimental data would be strictly protected. After completing the questionnaires, participants proceeded to the formal experimental environment.

**Figure 1:**
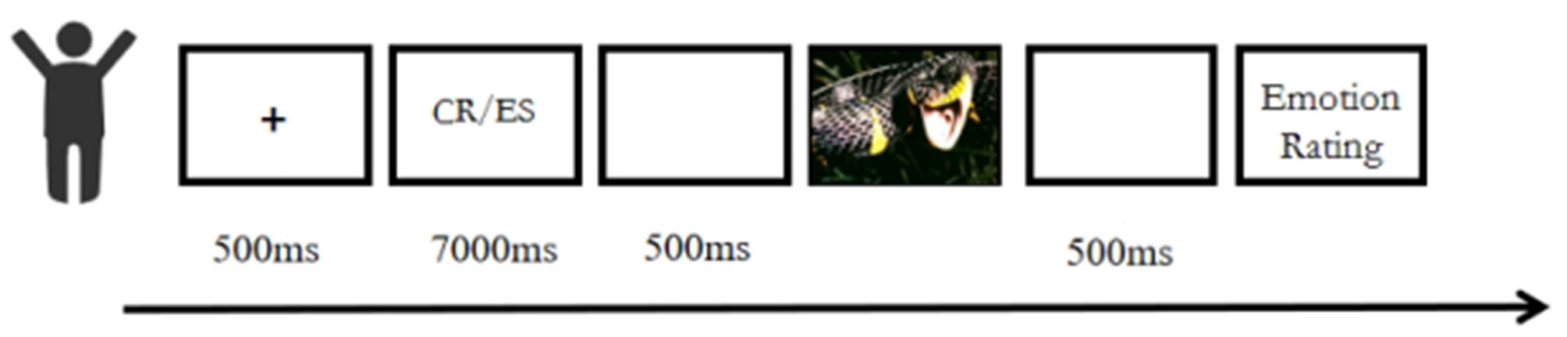
Flowchart of self-emotion regulation.

The experimental procedure was programmed using E-Prime 2.0, with reference to existing studies (Xu et al., 2021; Yao et al., 2025; Yao and Wang, 2023; Zhang et al., 2023; Cheng et al., 2011; Liu et al., 2023). The specific workflows for the self-emotion regulation group and the interpersonal emotion regulation group are as follows:

Self-Emotion Regulation Group: A fixation point was first presented for 500 ms, followed by a cognitive reappraisal or expressive suppression strategy sentence. After a 500 ms buffer screen, a negative emotion image was displayed for 5000 ms. Participants were then required to rate their current emotional state on a 9-point scale. The experiment consisted of 2 blocks (cognitive reappraisal and expressive suppression), including 5 practice trials. Each block contained 32 formal trials (the experimental workflow is shown in Figure 1).

**Figure 2:**
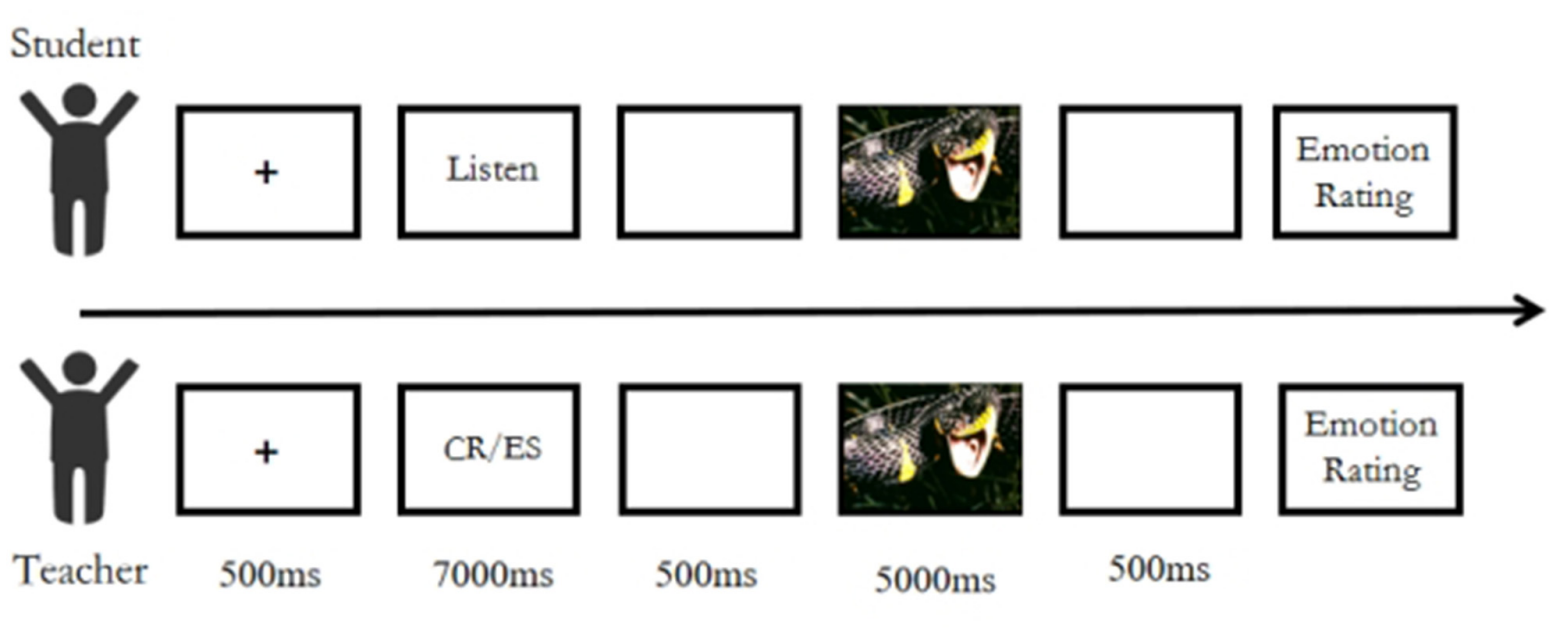
Flowchart of interpersonal emotion regulation.

**Figure 3:**
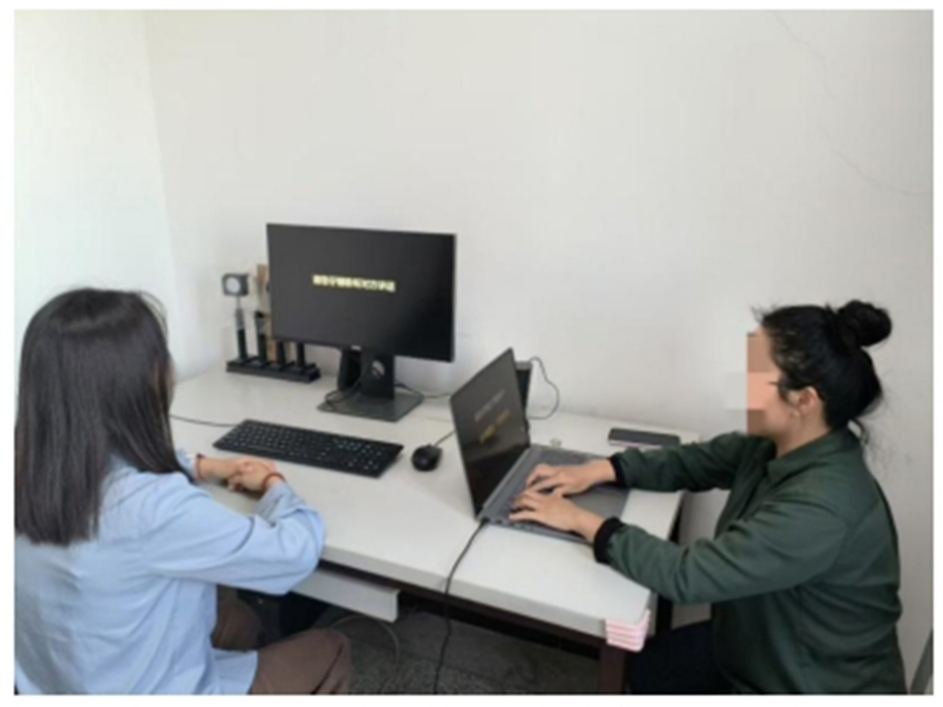
Experimental setup for interpersonal emotion regulation.

Interpersonal Emotion Regulation Group: Upon entering the laboratory, the paired participants were seated at a 90-degree angle to each other. A fixation point was first presented for 500 ms, followed by the display of a cognitive reappraisal or expressive suppression strategy sentence on the teacher's screen, who was instructed to read it aloud. Simultaneously, the student's screen displayed the prompt: “Please listen carefully to the speaker.” After a 500 ms buffer screen, a negative emotion image was presented for 5,000 ms. Both participants were then required to rate their current emotional state on a 9-point scale. The experiment also consisted of 2 blocks (cognitive reappraisal and expressive suppression), including 5 practice trials. Each block contained 32 formal trials, and the sequence of the 2 blocks was counterbalanced among participants (the experimental workflow is shown in Figure 2, and the experimental setup is illustrated in Figure 3).

### 2.5 Data analysis

The data statistical analysis was performed using SPSS 26.0. The analytical approach and methods were as follows: (1) *T-tests* were employed to assess whether there were significant differences in pre-experiment emotional states between the two groups of participants; (2) *T-tests* were employed to assess whether there were significant differences in habitual use of emotion regulation strategies between the two groups; (3) ANOVA was performed to analyze the 2 (interpersonal vs. self) × 2 (cognitive reappraisal vs. expressive suppression) mixed experimental design.

### 2.6 Results

#### 2.6.1 Data preprocessing

*T-tests* were employed on the PANAS scores completed by the participants. The results revealed no significant differences in pre-experiment emotional states between groups: for PA, *t* = –*1.44, p* = *0.15* > *0.05*; for NA, *t* = –*1.14, p* = *0.26* > *0.05*. These findings indicate that participants' emotional states did not differ significantly prior to the formal experiment.

*T-tests* were employed on the ERQ completed by the participants. The results showed no significant differences in the habitual use of emotion regulation strategies between groups: for CR, *t* = –*0.55, p* = *0.59* > *0.05*; for ES, *t* = *0.84, p* = *0.41* > *0.05*. These findings indicate that participants did not differ significantly in their habitual use of emotion regulation strategies.

#### 2.6.2 Effects of emotion regulation types and strategies on emotion regulation effectiveness in graduate students

**Figure 4:**
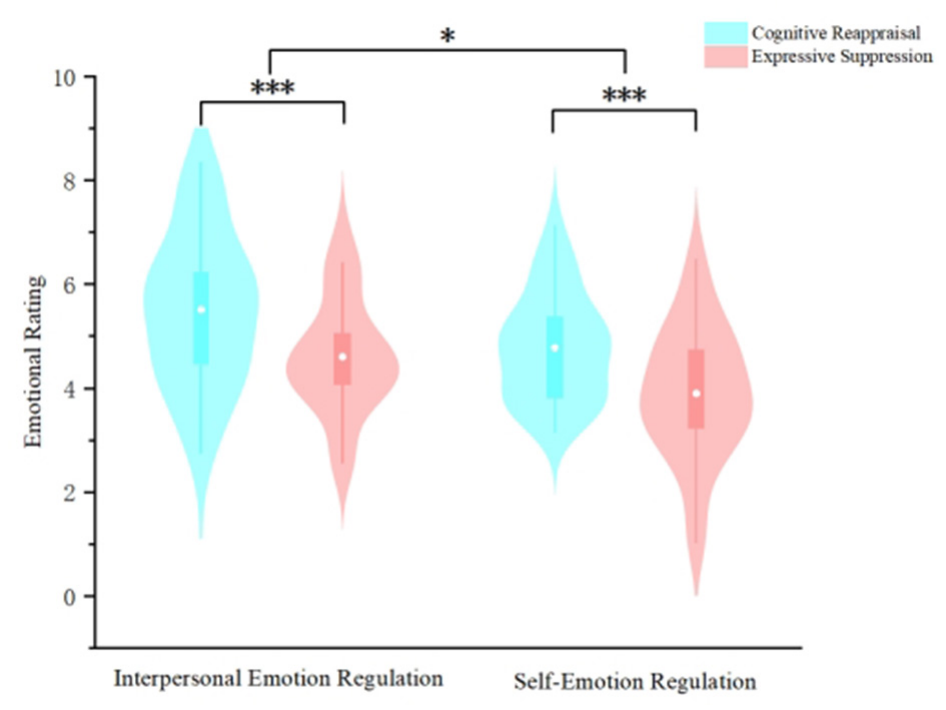
Effects of emotion regulation types and strategies on emotion regulation effectiveness in graduate students. (***p < 0.001,*p < 0.05).

ANOVA was conducted with emotion regulation type (self vs. interpersonal) and emotion regulation strategy (CR vs. ES) as independent variables, and self-reported emotional scores as the dependent variable (descriptive results are presented in Table 1). The results revealed that the interaction effect between emotion regulation type and emotion regulation strategy was not significant (*F*_(1,56)_ =*0.01, p* = *0.92* > *0.05*, η_*p*_^2^ < *0.001*). The main effect of emotion regulation type was significant (*F*_(1,56) =_ *6.04, p* = *0.017* < *0.05*, η_*p*_^2^ = *0.10*), with significantly higher emotional pleasantness under IER conditions compared to self-emotion regulation. The main effect of emotion regulation strategy was also significant (*F*_(1,56)_ = *43.83, p* < *0.001*, η_*p*_^2^ = *0.44*), with significantly higher emotional pleasantness under cognitive reappraisal strategies compared to expressive suppression strategies (as shown in Figure 4).

**Table 1:** Descriptive statistical analysis results of emotion regulation effectiveness under different conditions.

**Emotion regulation type**	**Emotion regulation strategy**	**Emotional rating (*M ±SD*)**
Interpersonal emotion regulation	Cognitive reappraisal	5.51 ± 1.46
	Expressive suppression	4.61 ± 1.11
Self-emotion regulation	Cognitive reappraisal	4.78 ± 1.04
	Expressive suppression	3.90 ± 1.28

### 2.7 Discussion

This research sought to investigate the impacts of emotion regulation types and strategies on emotion regulation effectiveness in graduate students. The results demonstrated that, across different emotion regulation strategies, IER was significantly superior to self-emotion regulation. This difference may stem from the advantages of IER in leveraging social support and external executive capabilities, which provide individuals with richer and more effective strategic resources. Consequently, it compensates for the limitations of self-regulation in terms of resources and perspectives. Through interpersonal interaction and external guidance, individuals can enhance their emotion management abilities, thereby promoting psychological wellbeing and social adaptation (Reeck et al., 2016).

When facing academic pressure, research challenges, or interpersonal conflicts, students often fall into affect-congruent thinking patterns. This cognitive mode not only potentially amplifies existing negative emotional responses (Elliott et al., 2002; Reeck et al., 2016) but may also impair prefrontal control system functions (Arnsten, 2009; Reeck et al., 2016). Supervisors, leveraging their substantial practical experience and deep understanding of students' developmental characteristics, can intervene from an external perspective to help students break this cognitive pattern and guide them out of emotional difficulties.

Based on the above findings, Study 2 will further focus on the influencing factors of interpersonal emotion regulation to deeply reveal its underlying mechanisms.

## 3 Study 2: the impact of teacher-student closeness and emotion regulation strategies on interpersonal emotion regulation effectiveness in graduate students

### 3.1 Participants

Sample size calculation was performed via G^*^Power 3.1 (Faul et al., 2009). With an *effect size* of *0.25*, α set to *0.05*, and *1-*β at *0.95*, the analysis showed 54 participants were needed. This study actually recruited 62 university teachers (including 30 postgraduate supervisors and 32 unfamiliar teachers; 25 males and 37 females, *M* ± *SD*_*age*_ =37.79 ± 6.82). Simultaneously, 62 graduate students were recruited (including 30 students advised by the aforementioned supervisors; 8 males and 54 females, *M* ± *SD*_*age*_ =24.79 ± 1.94). All involved individuals possessed normal vision or vision corrected to normal, with no neurological conditions present.

### 3.2 Measures

#### 3.2.1 Emotional picture stimuli

Identical to Study 1.

#### 3.2.2 Emotion regulation strategy sentence materials

Identical to Study 1.

#### 3.2.3 PANAS

Identical to Study 1. In this study, the Cronbach's α was 0.78 for the PA subscale and 0.87 for the NA subscale.

#### 3.2.4 Negative emotion rating scale

Identical to Study 1.

#### 3.2.5 Emotion Regulation Questionnaire (ERQ)

Identical to Study 1. In this study, the Cronbach's α was 0.76 for the cognitive reappraisal subscale and 0.79 for the expressive suppression subscale.

#### 3.2.6 Inclusion of Other in the Self Scale (IOS)

**Figure 5:**
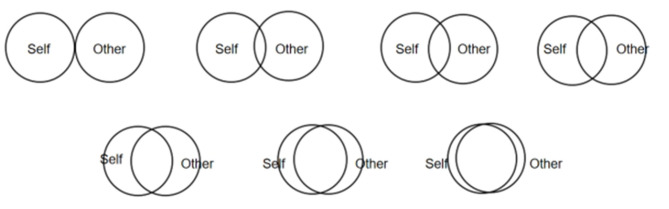
Inclusion of other in the self scale.

Derived from Berscheid et al. (1989) Relationship Closeness Inventory (RCI), this scale was developed by Aron et al. (1992). It gauges perceived closeness between two people by assessing the overlap of two circles, using a 7-point rating system—higher scores signify stronger relational closeness (Figure 5; Cao et al., 2018).

### 3.3 Experimental design

2 × 2 mixed experimental design was adopted, with emotion regulation strategy (CR vs. ES) as the within-subjects variable and teacher-student closeness (high vs. low) as the between-subjects variable. The high-closeness group consisted of supervisor-graduate student pairs, while the low-closeness group consisted of unfamiliar teacher-graduate student pairs. The dependent variable was the students' self-reported negative emotion ratings.

### 3.4 Experimental procedure

Prior to the formal experiment, preparatory procedures were completed. For the low-closeness group, unfamiliar teachers and students were randomly paired; for the high-closeness group, a supervisor, and one of their personally advised graduate students participated together. Upon arrival, participants were guided to separate rooms to complete questionnaires independently. During this process, the experimenter explained the confidentiality principle to all participants, ensuring strict protection of personal information and experimental data. After questionnaire completion, both parties entered the experimental room together.

The experiment included two conditions: low-closeness and high-closeness teacher-student groups. The experimental workflow was consistent with the interpersonal emotion regulation procedure described in Study 1.

### 3.5 Data analysis

The data statistical analysis was performed using SPSS 26.0. The analytical approach and methods were as follows: (1) *T-tests* were conducted to examine whether there were significant differences in participants‘ pre-experiment emotional states; (2) *T-tests* were employed to assess whether there were significant differences in participants' habitual use of emotion regulation strategies; (3) *T-tests* were performed to evaluate the effectiveness of the closeness grouping; (4) ANOVA was applied to analyse the 2 (CR vs. ES) × 2 (high closeness vs. low closeness) mixed experimental design.

### 3.6 Results

#### 3.6.1 Data preprocessing

*T-tests* were employed on the PANAS scores completed by the participants. The results showed no significant differences in pre-experiment emotional states: for PA, *t* = *1.94, p* = *0.06* > *0.05*; for NA, *t* = –*0.89, p* = *0.38* > *0.05*. These findings indicate that participants' emotional states did not differ significantly prior to the formal experiment.

*T-tests* were employed on the Emotion Regulation Questionnaire (ERQ) completed by the participants. The results showed no significant differences in the habitual use of emotion regulation strategies between groups: for CR, *t* = *1.43, p* = *0.16* > *0.05*; for ES, *t* = –*0.31, p* = *0.76* > *0.05*, indicating that participants did not differ significantly in their habitual use of emotion regulation strategies.

*T-tests* were employed on the Inclusion of Other in the Self (IOS) scale scores completed by the student participants. The results revealed a significant difference: *t* = *19.68, p* < *0.001*, indicating that the grouping into high- and low-closeness conditions was effective.

#### 3.6.2 The impact of teacher-student closeness and emotion regulation strategies on interpersonal emotion regulation effectiveness in graduate students

**Figure 6:**
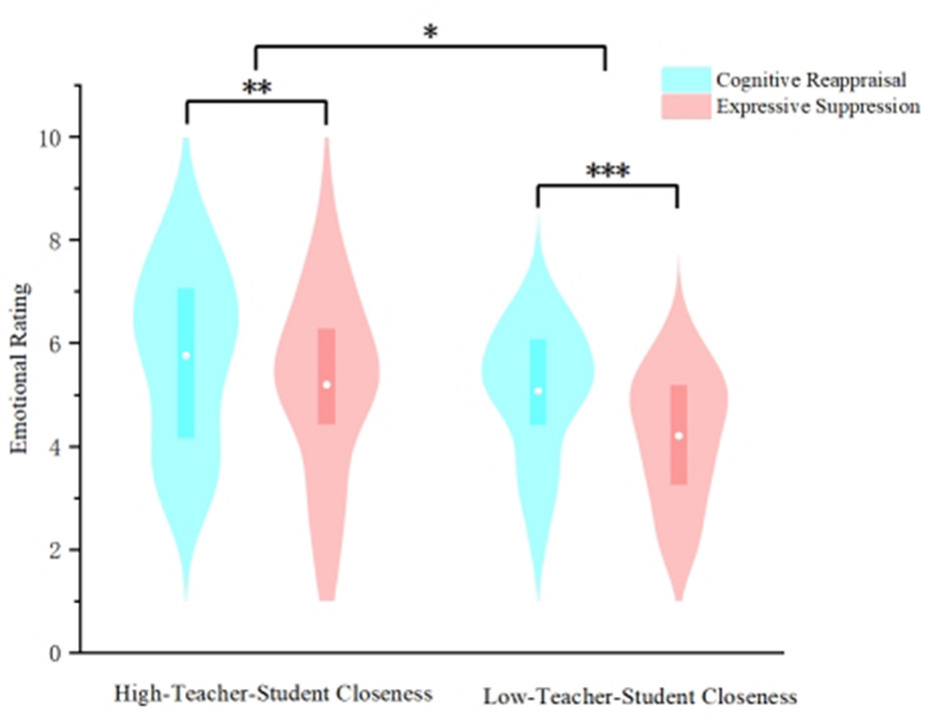
Effects of teacher-student closeness and emotion regulation strategies on interpersonal emotion regulation effectiveness in graduate students. (***p < 0.001, **p < 0.05,*p < 0.05).

ANOVA were employed with teacher-student closeness (high vs. low) and emotion regulation strategy (CR vs. ES) as independent variables, and self-reported emotional scores as the dependent variable (descriptive results are presented in Table 2). The results revealed that the interaction effect between teacher-student closeness and emotion regulation strategy was not significant (*F*_(1,60)_ = *0.54, p* = *0.47* > *0.05*, η_*p*_^2^ = *0.01*). The main effect of teacher-student closeness was significant (*F*_(1,60)_ = *4.28, p* = *0.04* < *0.05*, η_*p*_^2^ = *0.07*), with significantly higher emotional pleasantness among graduate students in the high-closeness group compared to the low-closeness group. The main effect of emotion regulation strategy was also significant (*F*_(1,60)_ = *35.34, p* < *0.001*, η_*p*_^2^ = *0.37*), demonstrating significantly higher emotional pleasantness under cognitive reappraisal strategies compared to expressive suppression strategies (as shown in Figure 6).

**Table 2:** Descriptive statistical analysis results of emotion regulation effectiveness under different conditions.

**Teacher-student closeness**	**Emotion regulation strategy**	**Emotional rating (*M ±SD*)**
High	Cognitive reappraisal	5.76 ± 1.72
	Expressive suppression	5.20 ± 1.81
Low	Cognitive reappraisal	5.07 ± 1.28
	Expressive suppression	4.34 ± 1.25

### 3.7 Discussion

This study investigated the effects of teacher-student closeness and emotion regulation strategies on interpersonal emotion regulation through behavioral experiments. The results revealed that closeness is a critical factor influencing the effectiveness of interpersonal emotion regulation.

These findings are consistent with existing research (Yao et al., 2025; Morawetz et al., 2021): individuals exhibit higher trust in support provided by friends, thereby achieving superior interpersonal emotion regulation effects compared to interactions with strangers (Morawetz et al., 2021). This study further extends these findings to educational contexts. Although teacher-student relationships differ in nature from friendships, they share common mechanisms through which trust and support enhance emotion regulation effectiveness. Social proximity can alter an individual's perception of their environment and provide supportive scaffolding for self-regulation capabilities. Simultaneously, the Social Baseline Theory also indicates that when individuals are near familiar and predictable others, their regulation of emotions and behaviors becomes more efficient (Beckes and Sbarra, 2022).

Close relationships contribute to enhancing mutual understanding between parties. For instance, friends are more accurate than strangers in comprehending the sender's intentions (Sui et al., 2024). They may also facilitate self-other integration at the cognitive level among interacting members (Quintard et al., 2020; Song et al., 2024). Furthermore, when facing negative emotions, individuals are more inclined to share with close others (Liu et al., 2021), further highlighting the critical role of intimate relationships in the emotion regulation process.

## 4 General discussion

This study investigated the advantageous effect of IER in educational contexts and its influencing factors through two behavioral experiments. The results revealed that, regardless of whether CR or ES strategies were employed, the effectiveness of IER was significantly superior to that of self-emotion regulation. Additionally, teacher-student closeness was identified as a key factor influencing the outcomes of IER.

First, this study demonstrates a stable advantageous effect of IER in educational contexts. This finding aligns with the common experience that “the observer sees more clearly than the participant”—when individuals are immersed in intense emotional experiences, they often struggle to objectively evaluate their own emotional states and behavioral responses. In contrast, external others, as “outsiders,” can provide novel cognitive perspectives and emotional interpretations, thereby facilitating the individual's understanding and management of emotions. Existing research also indicates that interpersonal emotion regulation holds advantages over self-regulation, primarily because external regulators offer a more objective perspective, effectively circumventing the cognitive biases and interference experienced by the emotionally involved individual (Levy-Gigi and Shamay-Tsoory, 2017; Franklin-Gillette and Shamay-Tsoory, 2021). This objective viewpoint from others contributes to enhancing the rationality and execution efficacy of emotion regulation.

Secondly, teacher-student closeness has been confirmed as a critical factor influencing the effectiveness of IER. According to the Person-Context Interaction Theory (Magnusson and Stattin, 1998), individual development is closely intertwined with their environment. In educational settings, teacher-student relationships serve as a key element of the school environment and play a vital role in students' emotional health development. The findings of this study, situated within the context of China's tutor responsibility system, are consistent with the global understanding of how the supervisor's role impacts graduate students' mental health. Studies across various countries have demonstrated the critical role of supervisors in promoting graduate students' mental health (Busch et al., 2024; Tenenbaum et al., 2001; Zhang Y. et al., 2024). Meanwhile, dysfunctional teacher-student relationships can impair students' psychological well-being (SenthilKumar et al., 2023). The consistent results of these cross-regional studies indicate that the issue of graduate students' mental health and its association with supervisor-related factors is not a unique phenomenon confined to a specific culture or institutional setting, but rather a universal concern that warrants collective attention within the global higher education system.

From the perspective of intimate relationships, existing studies have demonstrated that closeness significantly facilitates emotion regulation processes. For instance, physical contact with friends (e.g., hand-holding) effectively suppresses the visual cortex's processing of aversive stimuli, thereby reducing negative emotions (Kawamichi et al., 2015). Even the mere awareness of a friend participating in the same experiment in an adjacent room can enhance an individual's positive emotional experience (Yao et al., 2025). This effect may stem from close relationships enhancing individuals' perception of social cues and cognitive appraisal capabilities at the conscious level, enabling them to exhibit stronger conscious empathy toward high-closeness others and thus understand and respond to others' emotions more deeply (Song et al., 2016). As a form of close relationship, teacher-student relationships follow the same general principles through which intimacy influences emotion regulation. Whether the close relationship is built with friends or teachers, its essence revolves around stable interpersonal bonds and mutual support. Therefore, closeness plays a pivotal role in the process of IER, significantly shaping students' emotion regulation outcomes.

Furthermore, integrating the results of Study 1 and Study 2 reveals that CR consistently outperforms ES across different contexts. These findings align with previous research (Goldin et al., 2008; Ma et al., 2010; Cheng et al., 2011). During social interactions, cognitive reappraisal helps individuals more accurately recognize others' negative emotions while serving an “emotional protective” function that effectively reduces one's own experience of negative affect (Guo et al., 2023). In contrast, although expressive suppression can reduce the expression of negative emotions to some extent, it is fundamentally an avoidance or suppression strategy. Not only does it fail to alleviate negative emotions effectively, but it may also incur subsequent adaptive costs due to emotional rebound effects (Zhang S. et al., 2024).

On one hand, this study breaks through the single framework of family/peer relationships in traditional interpersonal emotion regulation research by focusing for the first time on teacher-student relationships within the educational ecosystem, thereby enriching the research subjects of IER. On the other hand, it innovatively explores the advantageous effects of IER in educational contexts, providing new directions for theoretical applications in this field. In terms of practical value, the study offers scientific methods for university educators to guide them in scientifically constructing and effectively maintaining positive teacher-student relationships. By optimizing the quality of teacher-student interactions, it tangibly promotes the emotional health development of graduate students.

This study has several limitations: First, in terms of emotion regulation strategies, the research focused solely on two typical strategies—CR and ES—without encompassing other types of emotion regulation strategies. Second, the gender distribution of the participant population was uneven. Future studies should strive to balance and control the gender ratio of participants. Additionally, the current analysis of the advantageous effects and influencing factors of interpersonal emotion regulation was conducted only at the behavioral level, without delving into the underlying neural mechanisms. Future research could incorporate neuroimaging techniques to explore the neural mechanisms of interpersonal emotion regulation. Fourth, the model validation and findings of this study are primarily based on Chinese graduate student samples and the Chinese cultural-educational context. We acknowledge that specific cultural backgrounds (e.g., respect for authority, collectivistic orientation) and institutional arrangements (e.g., the “tutor responsibility system”) may render certain aspects of the research findings context-specific. However, it is important to note that in the literature review and theoretical construction of this study, we intentionally incorporated studies conducted in China and published in international journals. These studies themselves contribute to the global discourse on “teacher-student interaction and mental health,” and their value lies in providing internationally recognized, high-quality, and dialoguable empirical evidence. Future research urgently needs to disentangle and examine the specific role of cultural traits through systematic cross-cultural comparisons (e.g., comparing individualistic and collectivistic cultures), which will greatly enhance the external validity and explanatory scope of the theory. Finally, this study was mainly conducted in laboratory settings, and there are certain differences between experimental and naturalistic contexts, which may raise questions about the applicability of the research results in real-world scenarios. Future studies should focus on integrating naturalistic contexts and adopting more ecologically valid research methods to improve the ecological validity of the findings.

## 5 Conclusions

(1) In educational contexts, interpersonal emotion regulation demonstrates a significant advantageous effect; (2) Higher teacher-student closeness leads to stronger effectiveness of interpersonal emotion regulation among graduate students; (3) The emotion regulation effectiveness of cognitive reappraisal strategies is significantly superior to that of expressive suppression strategies.

In summary, against the backdrop of China's unique educational ecology and cultural context, this study reveals the positive pathways through which supervisors' interpersonal emotion regulation influences graduate students' mental health. Despite the context-specificity of the research setting, the theoretical perspectives, empirical findings, and methodological explorations presented herein hold implications for broader geographical and cultural regions. Researchers in other parts of the world, when addressing the shared challenge of enhancing students' mental health through optimizing teacher-student interactions, may draw on the comparative framework, measurement insights, or intervention design inspiration offered by this study. We believe that only by accumulating evidence from diverse cultural backgrounds and engaging in sincere academic dialogue can we construct a more inclusive and explanatory global knowledge system, thereby addressing this pervasive concern in higher education.

## Data Availability

The raw data supporting the conclusions of this article will be made available by the authors, without undue reservation.
